# Experimental data from the simulation of on-chip communication architectures using *RedScarf* simulation environment

**DOI:** 10.1016/j.dib.2019.104725

**Published:** 2019-10-28

**Authors:** Eduardo Alves da Silva, Márcio Eduardo Kreutz, Cesar Albenes Zeferino

**Affiliations:** a University of Vale do Itajaí – Univali, Brazil; b Federal University of Rio Grande do Norte – UFRN, Brazil

**Keywords:** Multi- and many-core systems, Network-on-Chip, Performance evaluation, Simulation

## Abstract

This article presents data from an extensive set of simulation-based experiments to compare the performance of on-chip communication architectures. These experiments were performed using the *RedScarf* simulation environment [1], which is described in the article entitled ‘*RedScarf*: an open-source multi-platform simulation environment for performance evaluation of Networks-on-Chip’ [2]. In the experiments presented here, several intra-chip communication architectures were compared under different traffic patterns. Latency, jitter, and throughput metrics were collected. Data is useful for researchers investigating on-chip communication architectures who need baseline data for comparison.

**Table:** Specifications Table

Subject	Hardware and Architecture
Specific subject area	Simulation-based performance evaluation of on-chip communication architectures
Type of data	Tables
How data were acquired	Computational simulation
Data format	Raw and analyzed
Parameters for data collection	Experiments are based on traffic patterns composed of communication flows configured to inject 128-bit packets into 32-bit data links at a constant injection rate of 320 Mbps.
Description of data collection	Data were obtained using modules which collect information about each packet delivered by the communication architecture. These modules collect the necessary information to calculate the performance metrics, which are: the average latency, the jitter, and the throughput.
Data source location	University of Vale do Itajaí – Univali, Itajaí, Brazil
Data accessibility	Analyzed data with the article Raw data available at the following repository: Mendeley Data: https://doi.org/10.17632/wy9y7npwyv.1 Repository name: RedScarf Reference Dataset
Related research article	E.A. Silva, M.E. Kreutz, C.A. Zeferino, *RedScarf*: an open-source multi-platform simulation environment for performance evaluation of Networks-on-Chip, *Journal of Systems Architecture.* 99 (2019) 101633. doi: 10.1016/j.sysarc.2019.101633

**Table:** 

**Value of the Data** • Data were obtained from simulations experiments and can be useful for researchers and students to carry out comparative analysis concerning architectural configurations. • Data allow setting up experiments with different architectural configurations of communication mechanisms in Networks-on-Chip – NoCs. • Data provide a comparison among strategies during the design process of NoCs. • Data is pertinent to verify how a single change in an architectural parameter impacts performance over a range of communication behavior. • Data presented may instigate researchers to evaluate the efficiency of their approach to comply with design constraints for on-chip communication architectures.

## 1 Data

Table 1, Table 2, Table 3 present data obtained from experiments carried out using *RedScarf* [[1], [2]] to evaluate the performance of 64-node on-chip communication architectures submitted to six traffic patterns based on different spatial distributions. The tables present the average latency, the jitter, and the throughput measured by varying the operating frequency. The first metric is the average of the latency of all the packets delivered, while the second metric measures the dispersion of the latency suffered by these packets (i.e., the standard deviation). The third metric consists of the traffic that the network accepted given the offered traffic, which varies with the changing of the operating frequency because the injection rate is constant (320 Mbps). Table 4, Table 5, Table 6 show data related to the simulation of a 4 × 4 2D Mesh topology running with five different routing algorithms, including one deterministic algorithm and four partially-adaptive routing algorithms based on the Turn Model [3]. Table 7, Table 8, Table 9 presents data collected from experiments that evaluate the impact of four arbitration policies on the average latency, jitter, and throughput of a 4 × 4 Torus topology. Finally, Table 10, Table 11, Table 12 show the impact of the buffers depth and the use of output buffers on the three performance metrics of a 4 × 2 × 2 Mesh topology. Seven different memory schemes are employed.

**Table 1:** Average latency (in cycles) for 64-node on-chip network topologies under different traffic patterns.

Traffic Pattern	Network Topology	Operating Frequency (MHz)|Offered traffic (normalized)									
		10|1.00	20|0.50	30|0.33	40|0.25	50|0.20	60|0.16	70|0.14	80|0.12	90|0.11	100|0.10
Uniform	Bus	415,558.7	412,259.4	408,987.4	405,700.9	402,439.7	399,154.7	395,887.4	392,599.2	389,319.2	386,041.3
	2D Mesh	18,208.3	14,574.9	10,812.5	7302.1	5480.8	4429.9	3739.5	2596.6	2101.1	1595.0
	3D Mesh	9456.0	6044.3	2794.0	739.4	525.9	321.2	286.3	237.6	221.8	213.3
	Crossbar	9311.1	6400	3252.2	1020.4	750.3	247.3	214.1	203.3	193.0	181.4
Bit-reversal	2D Mesh	32,873.9	25,019.2	19,164.6	13,944.9	12,348.1	9145	9060.7	8043.4	6953.3	5702.3
	3D Mesh	17,308.7	12,782.5	8256.2	5070.9	3707.7	2145.1	1224.3	21.8	21.8	21.8
	2D Torus	25,600.2	20,779.5	15,958.8	11,319.1	7771.1	5370	2559.3	463.7	308.1	27.9
	Crossbar	2506.0	7.0	7.0	7.0	7.0	7.0	7.0	7.0	7.0	7.0
Perfect Shuffle	Chordal Ring	58,529.1	37,603.6	37,207.3	33,684.1	33,311.4	31,718.0	34,424.7	36,611.7	27,195.2	23,669.5
	2D Mesh	14,537.6	10,934.0	8874.9	6945	5911.9	4140.7	2090.1	24.2	24.2	24.2
	3D Mesh	6024.5	3255.7	1617.1	17.7	17.7	17.7	17.7	17.7	17.7	17.7
	2D Torus	10,488.6	7180.8	5114.7	3152.1	2370.6	25.1	24.4	23	23	23
	Crossbar	2264.0	7.0	7.0	7.0	7.0	7.0	7.0	7.0	7.0	7.0
Butterfly	Chordal Ring	4387.0	13.0	13.0	13.0	13.0	13.0	13.0	13.0	13.0	13.0
	2D Mesh	40,485.4	31,464.4	22,443.4	15,923.2	12,330.6	8085.1	4002.8	26.5	26.5	26.5
	3D Mesh	17,513.5	10,952.5	4391.5	17.5	17.5	17.5	17.5	17.5	17.5	17.5
	2D Torus	40,485.4	31,464.4	22,443.4	15,923.2	12,330.6	8085.1	4002.8	26.5	26.5	26.5
	Crossbar	4381.0	7.0	7.0	7.0	7.0	7.0	7.0	7.0	7.0	7.0
Transpose	2D Mesh	20,804.5	15,233.8	11,084.2	7777.3	6499.9	4890.0	4258.0	3208.8	2775.1	1962.3
	3D Mesh	21,224.1	16,282.7	12,391.2	10,057.8	6874.7	5046.9	2461.2	314.5	25.3	25.1
	2D Torus	19,973.3	15,258.8	10,544.2	6937.3	4463.7	2147.5	1227.1	24.6	24.6	24.6
	Crossbar	2506.0	7.0	7.0	7.0	7.0	7.0	7.0	7.0	7.0	7.0
Complement	Chordal Ring	40,839.9	32,091.8	23,343.7	17,286.5	14,597.9	11,831.7	10,605.8	8972.9	8286.3	7140
	2D Mesh	44,238.6	38,037.5	31,836.3	25,716.2	19,664.5	14,299.8	9593.1	40.0	40.0	40.0
	3D Mesh	8773.5	5494.5	2215.5	29.5	29.5	29.5	29.5	29.5	29.5	29.5
	2D Torus	8768.0	5488.2	2208.5	22.0	22.0	22.0	22.0	22.0	22.0	22.0
	Crossbar	2194.0	7.0	7.0	7.0	7.0	7.0	7.0	7.0	7.0	7.0

**Table 2:** Jitter for 64-node on-chip network topologies under different traffic patterns.

Traffic Pattern	Network Topology	Operating Frequency (MHz)|Offered traffic (normalized)									
		10|1.00	20|0.50	30|0.33	40|0.25	50|0.20	60|0.16	70|0.14	80|0.12	90|0.11	100|0.10
Uniform	Bus	102,933.8	102,151.5	101,321.5	100,525.5	99,694.3	98,889.6	98,055.5	97,255.7	96,449.4	95,629.5
	2D Mesh	4609.9	4371.3	4588.6	5097.8	5164.2	4692.9	4279.1	3660.5	3216.6	2692.4
	3D Mesh	2225.3	1531.3	1017.5	695.0	686.9	292.2	293.2	185.3	166.9	162.3
	Crossbar	2058.8	1318.2	939.1	966.8	1028.4	173.3	138.2	130.3	124.4	109.5
Bit-reversal	2D Mesh	9428.0	10,541.0	12,834.3	14,470.9	14,920.7	13,272.3	13,896.1	12,542.0	11,634.0	10,245.7
	3D Mesh	4792.7	4918.8	5757.6	6220.6	5194.6	4561.5	2168.7	7.7	7.7	7.7
	2D Torus	6943.3	7157.4	8137.6	9259.6	8292.7	7120.4	5728.0	1864.1	1107.5	7.2
	Crossbar	618.9	0.0	0.0	0.0	0.0	0.0	0.0	0.0	0.0	0.0
Perfect Shuffle	Chordal Ring	29,759.4	41,351.0	45,261.4	50,348.4	51,298.8	54,240.2	52,370.6	49,845.8	49,562.7	51,798.0
	2D Mesh	5556.9	6670.0	6418.7	6800.0	6677.4	5732.1	2832.1	8.4	8.4	8.4
	3D Mesh	2359.0	2494.0	1054.4	4.7	4.7	4.7	4.7	4.7	4.7	4.7
	2D Torus	4051.4	4755.3	4423.2	4294.5	3227.9	10	8.9	7.1	7.1	7.1
	Crossbar	558.9	0.0	0.0	0.0	0.0	0.0	0.0	0.0	0.0	0.0
Butterfly	Chordal Ring	1082.6	0.0	0.0	0.0	0.0	0.0	0.0	0.0	0.0	0.0
	2D Mesh	11,600.2	12,865.4	15,653.0	16,698.5	13,597.7	11,050.0	4592.2	3.4	3.4	3.4
	3D Mesh	4330.1	2706.3	1082.5	1.5	1.5	1.5	1.5	1.5	1.5	1.5
	2D Torus	11,600.2	12,865.4	15,653.0	16,698.1	13,601.9	11,049.5	4591.9	3.4	3.4	3.4
	Crossbar	1082.6	0.0	0.0	0.0	0.0	0.0	0.0	0.0	0.0	0.0
Transpose	2D Mesh	7522.4	8959.4	9554.7	10,372.3	9433.4	9335.1	8402.9	8079.1	7066.0	6489.2
	3D Mesh	6235.2	7129.5	8027.0	7894.8	7683.6	6805.7	4615.3	2190.3	8.2	8.3
	2D Torus	5597.2	5950.4	7069.6	7266.1	5354.8	4562.9	2169.9	9.3	9.3	9.3
	Crossbar	618.9	0.0	0.0	0.0	0.0	0.0	0.0	0.0	0.0	0.0
Complement	Chordal Ring	11,973.2	13,759.6	17,061.4	18,905.5	17,575.3	17,552.6	16,282.8	16,018.8	14,892.2	14,495.5
	2D Mesh	12,484.5	14,102.1	16,944.8	17,607.8	15,314.9	13,574.6	10,812.8	14.2	14.2	14.2
	3D Mesh	2166.2	1353.9	541.6	7.8	7.8	7.8	7.8	7.8	7.8	7.8
	2D Torus	2166.2	1353.9	541.6	6.4	6.4	6.4	6.4	6.4	6.4	6.4
	Crossbar	541.6	0.0	0.0	0.0	0.0	0.0	0.0	0.0	0.0	0.0

**Table 3:** Throughput (normalized) for 64-node on-chip network topologies under different traffic patterns.

Traffic Pattern	Network Topology	Operating Frequency (MHz)|Offered traffic (normalized)									
		10|1.00	20|0.50	30|0.33	40|0.25	50|0.20	60|0.16	70|0.14	80|0.12	90|0.11	100|0.10
Uniform	Bus	0.0074	0.0074	0.0074	0.0074	0.0074	0.0074	0.0074	0.0074	0.0074	0.0074
	2D Mesh	0.1439	0.1441	0.1432	0.1443	0.1334	0.1239	0.1127	0.1049	0.0975	0.0906
	3D Mesh	0.2418	0.2432	0.2451	0.2187	0.1807	0.1513	0.1298	0.1142	0.1018	0.0920
	Crossbar	0.2558	0.2542	0.2475	0.2171	0.1798	0.1512	0.1302	0.1142	0.1018	0.0921
Bit-reversal	2D Mesh	0.0773	0.0773	0.0773	0.0766	0.0729	0.0762	0.0702	0.0701	0.0684	0.0675
	3D Mesh	0.1404	0.1404	0.1404	0.1368	0.1285	0.1216	0.1135	0.1049	0.0937	0.0846
	2D Torus	0.1022	0.1022	0.1022	0.1024	0.1039	0.1029	0.1035	0.1030	0.0928	0.0846
	Crossbar	0.4374	0.3750	0.2625	0.2019	0.1641	0.1382	0.1193	0.1050	0.0937	0.0847
Perfect Shuffle	Chordal Ring	0.0188	0.0174	0.0150	0.0137	0.0129	0.0123	0.0119	0.0116	0.0114	0.0112
	2D Mesh	0.1548	0.1503	0.1424	0.1325	0.1228	0.1205	0.1203	0.1161	0.1037	0.0937
	3D Mesh	0.2963	0.2809	0.2527	0.2233	0.1815	0.1528	0.1320	0.1161	0.1037	0.0937
	2D Torus	0.1994	0.1927	0.1810	0.1711	0.1556	0.1528	0.1320	0.1161	0.1037	0.0937
	Crossbar	0.4843	0.4151	0.2906	0.2235	0.1816	0.1530	0.1321	0.1162	0.1038	0.0937
Butterfly	Chordal Ring	0.2500	0.2143	0.1500	0.1154	0.0937	0.0789	0.0682	0.0600	0.0536	0.0484
	2D Mesh	0.0624	0.0624	0.0624	0.0624	0.0625	0.0625	0.0625	0.0600	0.0536	0.0484
	3D Mesh	0.1250	0.1250	0.1250	0.1154	0.0937	0.0789	0.0682	0.0600	0.0536	0.0484
	2D Torus	0.0624	0.0624	0.0624	0.0624	0.0625	0.0625	0.0625	0.0600	0.0536	0.0484
	Crossbar	0.2500	0.2143	0.1005	0.1154	0.0937	0.0789	0.0682	0.0600	0.0536	0.0484
Transpose	2D Mesh	0.1076	0.1056	0.1020	0.0983	0.0948	0.0913	0.0873	0.0839	0.0800	0.0763
	3D Mesh	0.1176	0.1176	0.1156	0.1104	0.1085	0.1055	0.1057	0.1029	0.0937	0.0846
	2D Torus	0.1258	0.1248	0.1248	0.1248	0.1247	0.1216	0.1135	0.1049	0.0937	0.0846
	Crossbar	0.4374	0.3750	0.2625	0.2019	0.1641	0.1382	0.1193	0.1050	0.0937	0.0847
Complement	Chordal Ring	0.0614	0.0614	0.0614	0.0620	0.0613	0.0615	0.0619	0.0617	0.0620	0.0621
	2D Mesh	0.0622	0.0622	0.0622	0.0646	0.0699	0.0755	0.0813	0.1199	0.1070	0.0967
	3D Mesh	0.2497	0.2497	0.2497	0.2305	0.1874	0.1578	0.1363	0.1199	0.1071	0.0967
	2D Torus	0.2498	0.2498	0.2498	0.2306	0.1874	0.1578	0.1363	0.1199	0.1071	0.0967
	Crossbar	0.4999	0.4285	0.3000	0.2308	0.1875	0.1579	0.1364	0.1200	0.1071	0.0968

**Table 4:** Average latency (in cycles) for a 4 × 4 Mesh using five routing algorithms under different traffic patterns.

Traffic Pattern	Routing Algorithm	Operating Frequency (MHz)|Offered traffic (normalized)									
		10|1.00	20|0.50	30|0.33	40|0.25	50|0.20	60|0.16	70|0.14	80|0.12	90|0.11	100|0.10
Uniform	XY	29,535.3	15,980.5	3302.5	83.3	59.6	51.9	47.6	45.0	43.3	42.2
	West-first	41,575.4	28,177.3	14,842.3	3823.4	584.4	63.7	53.1	47.8	44.9	43.2
	Negative-first	43,335.1	29,730.5	15,594.2	6463.9	3869.1	1836.9	75.9	52.8	48.2	45.3
	North-last	40,203.4	26,812.4	13,125.1	3184.8	88.9	62.9	53.7	48.9	45.6	43.7
	Odd-Even	41,654.8	28,264.3	14,217.3	6510.1	3493.3	1327.0	57.2	49.0	45.8	43.9
Bit-reversal	XY	58,815.4	39,916.7	21,018.0	8303.8	4863.5	20.5	20.5	20.5	20.5	20.5
	West-first	48,541.5	25,473.7	7607.8	2303.7	18.7	18.7	18.7	18.7	18.7	18.7
	Negative-first	44,045.6	23,555.9	152,72.6	8302.4	4862.2	19.2	19.2	19.2	19.2	19.2
	North-last	37,512.2	25,371.6	7111.0	3565.8	2303.7	18.7	18.7	18.7	18.7	18.7
	Odd-Even	35,709.2	17,033.0	3142.1	19.3	19.0	19.0	19.0	19.0	19.0	19.0
Perfect Shuffle	XY	19,612.7	8387.5	4861.6	14.7	14.7	14.7	14.7	14.7	14.7	14.7
	West-first	18,338.7	6674.1	2819.5	14.5	14.5	14.5	14.5	14.5	14.5	14.5
	Negative-first	18,592.8	6439.8	3571.9	14.5	14.5	14.5	14.5	14.5	14.5	14.5
	North-last	18,675.5	7369.8	2819.9	14.5	14.5	14.5	14.5	14.5	14.5	14.5
	Odd-Even	18,781.1	6045.1	2795.1	15.8	15.6	15.6	15.6	15.6	15.6	15.6
Butterfly	XY	70,013.5	43,765.0	17,516.5	17.5	17.5	17.5	17.5	17.5	17.5	17.5
	West-first	44,811.8	11,893.9	7247.5	16.8	16.8	16.8	16.8	16.8	16.8	16.8
	Negative-first	70,013.5	43,765.0	17,516.5	17.5	17.5	17.5	17.5	17.5	17.5	17.5
	North-last	44,811.8	11,893.9	7247.5	16.8	16.8	16.8	16.8	16.8	16.8	16.8
	Odd-Even	44,811.8	11,893.9	7247.5	16.8	16.8	16.8	16.8	16.8	16.8	16.8
Transpose	XY	40,845.5	25,204.1	16,842.1	8301.8	4861.8	19.0	19.0	19.0	19.0	19.0
	West-first	35,943.0	16,817.6	7448.3	3565.8	2303.2	18.2	18.2	18.2	18.2	18.2
	Negative-first	40,845.5	25,204.1	16,842.1	8301.8	4861.8	19.0	19.0	19.0	19.0	19.0
	North-last	35,943.0	16,817.6	7448.3	3565.8	2303.2	18.2	18.2	18.2	18.2	18.2
	Odd-Even	42,047.0	22,442.1	5339.3	18.7	18.2	18.2	18.2	18.2	18.2	18.2
Complement	XY	35,017.0	21,893.9	8770.8	22.0	22.0	22.0	22.0	22.0	22.0	22.0
	West-first	93,472.1	79,715.4	65,564.6	48,797.2	33,940.3	20,476.5	5193.2	27.4	25.7	27.7
	Negative-first	93,481.9	75,754.5	57,578.6	45,654.0	38,454.1	18,004.6	5551.1	6259.9	7015.5	26.1
	North-last	70,491.9	56,420.2	42,273.0	28,474.1	16,905.0	8842.5	4745.7	25.8	25.0	25.0
	Odd-Even	78,277.4	63,647.3	48,655.8	33,829.2	21,728.0	13,128.1	4349.8	26.8	26.4	26.9

**Table 5:** Jitter for a 4 × 4 Mesh using five routing algorithms under different traffic patterns.

Traffic Pattern	Routing Algorithm	Operating Frequency (MHz)|Offered traffic (normalized)									
		10|1.00	20|0.50	30|0.33	40|0.25	50|0.20	60|0.16	70|0.14	80|0.12	90|0.11	100|0.10
Uniform	XY	7368.1	4407.8	2539.3	60.5	35.7	30.9	27.9	26.1	25.1	24.4
	West-first	10,615.1	8546.1	7775.5	6046.2	1364.6	47.6	34.5	29.9	27.3	25.9
	Negative-first	11,456.6	10,255.6	10,914.0	10,053.6	8694.5	4278.6	91.8	37.5	31.8	28.9
	North-last	10,095.0	7405.2	5865.5	3786.9	71.1	39.9	34.3	30.6	27.9	26.4
	Odd-Even	11,014.6	9983.5	10,849.0	9884.2	7889.3	3954.0	43.3	31.1	27.9	26.4
Bit-reversal	XY	15,651.9	14,121.6	15,331.1	15,937.1	7825.1	5.5	5.5	5.5	5.5	5.5
	West-first	17,519.8	19,528.9	13,110.2	10,736.0	5797.0	5.1	5.1	5.1	5.1	5.1
	Negative-first	16,365.8	18,844.6	17,177.6	15,930.5	7825.9	5.5	5.5	5.5	5.5	5.5
	North-last	13,669.3	18,806.4	11,654.9	10,735.9	5797.0	5.1	5.1	5.1	5.1	5.1
	Odd-Even	10,614.4	11,285.1	6450.6	4.7	3.5	3.5	3.5	3.5	3.5	3.5
Perfect Shuffle	XY	8570.3	10,001.0	4888.5	3.9	3.9	3.9	3.9	3.9	3.9	3.9
	West-first	7707.8	8457.1	4300.4	3.4	3.4	3.4	3.4	3.4	3.4	3.4
	Negative-first	7939.3	8709.8	4752.0	3.4	3.4	3.4	3.4	3.4	3.4	3.4
	North-last	8164.0	9034.7	4300.3	3.4	3.4	3.4	3.4	3.4	3.4	3.4
	Odd-Even	4410.8	8711.8	7974.0	3.5	3.4	3.4	3.4	3.4	3.4	3.4
Butterfly	XY	17,320.6	10,825.4	4330.1	1.5	1.5	1.5	1.5	1.5	1.5	1.5
	West-first	17,954.4	16,285.9	8354.8	1.3	1.3	1.3	1.3	1.3	1.3	1.3
	Negative-first	17,320.6	10,825.4	4330.1	1.5	1.5	1.5	1.5	1.5	1.5	1.5
	North-last	17,954.4	16,285.9	8354.8	1.3	1.3	1.3	1.3	1.3	1.3	1.3
	Odd-Even	17,954.4	16,285.9	8354.8	1.3	1.3	1.3	1.3	1.3	1.3	1.3
Transpose	XY	16,813.0	19,790.0	16,759.5	15,940.0	7827.2	6.7	6.7	6.7	6.7	6.7
	West-first	13,003.4	14,728.7	11,537.3	10,734.8	5797.4	6.1	6.1	6.1	6.1	6.1
	Negative-first	16,813.0	19,789.2	16,759.1	15,942.4	7827.0	6.7	6.7	6.7	6.7	6.7
	North-last	13,003.4	14,728.7	11,537.3	10,734.8	5797.4	6.1	6.1	6.1	6.1	6.1
	Odd-Even	13,154.9	15,087.2	11,482.4	5.7	5.9	5.9	5.9	5.9	5.9	5.9
Complement	XY	8660.9	5413.1	2165.2	6.4	6.4	6.4	6.4	6.4	6.4	6.4
	West-first	23,909.9	22,302.5	29,077.4	23,295.1	25,763.6	25,160.3	10,419.8	9.8	6.7	8.0
	Negative-first	26,157.7	29,587.8	29,498.6	32,015.6	32,665.8	22,768.8	16,703.3	18,948.7	21,254.2	7.7
	North-last	18,004.0	16,084.2	15,477.2	16,524.8	17,128.8	13,797.7	9479.7	7.2	7.2	7.2
	Odd-Even	20,441.4	19,460.3	20,481.3	23,077.2	20,796.1	16,241.8	8325.0	7.7	6.4	7.4

**Table 6:** Throughput (normalized) for a 4 × 4 Mesh using five routing algorithms under different traffic patterns.

Traffic Pattern	Routing Algorithm	Operating Frequency (MHz)|Offered traffic (normalized)									
		10|1.00	20|0.50	30|0.33	40|0.25	50|0.20	60|0.16	70|0.14	80|0.12	90|0.11	100|0.10
Uniform	XY	0.2796	0.2804	0.2789	0.2306	0.1873	0.1577	0.1362	0.1198	0.1070	0.0966
	West-first	0.2208	0.2194	0.2216	0.2139	0.1856	0.1577	0.1361	0.1198	0.1070	0.0966
	Negative-first	0.2119	0.2117	0.2115	0.2004	0.1748	0.1536	0.1361	0.1198	0.1070	0.0966
	North-last	0.2275	0.2286	0.2277	0.2182	0.1871	0.1577	0.1362	0.1198	0.1070	0.0966
	Odd-Even	0.2186	0.2152	0.2146	0.2007	0.1769	0.1550	0.1361	0.1198	0.1070	0.0966
Bit-reversal	XY	0.1562	0.1562	0.1562	0.1490	0.1328	0.1184	0.1023	0.0900	0.0803	0.0726
	West-first	0.1631	0.1761	0.1874	0.1610	0.1367	0.1184	0.1023	0.0900	0.0803	0.0726
	Negative-first	0.1812	0.1830	0.1650	0.1490	0.1328	0.1184	0.1023	0.0900	0.0803	0.0726
	North-last	0.2014	0.1762	0.1796	0.1610	0.1367	0.1184	0.1023	0.0900	0.0803	0.0726
	Odd-Even	0.2155	0.2164	0.2108	0.1730	0.1406	0.1184	0.1023	0.0900	0.0803	0.0726
Perfect Shuffle	XY	0.3124	0.2856	0.2374	0.2019	0.1640	0.1381	0.1193	0.1050	0.0937	0.0847
	West-first	0.3222	0.2993	0.2468	0.2019	0.1640	0.1381	0.1193	0.1050	0.0937	0.0847
	Negative-first	0.3218	0.3012	0.2431	0.2019	0.1640	0.1381	0.1193	0.1050	0.0937	0.0847
	North-last	0.3175	0.2922	0.2468	0.2019	0.1640	0.1381	0.1193	0.1050	0.0937	0.0847
	Odd-Even	0.3095	0.2990	0.2468	0.2019	0.1640	0.1381	0.1193	0.1050	0.0937	0.0847
Butterfly	XY	0.1250	0.1250	0.1250	0.1154	0.0937	0.0789	0.0682	0.0600	0.0536	0.0484
	West-first	0.1562	0.1696	0.1375	0.1154	0.0937	0.0789	0.0682	0.0600	0.0536	0.0484
	Negative-first	0.1250	0.1250	0.1250	0.1154	0.0937	0.0789	0.0682	0.0600	0.0536	0.0484
	North-last	0.1562	0.1696	0.1375	0.1154	0.0937	0.0789	0.0682	0.0600	0.0536	0.0484
	Odd-Even	0.1562	0.1696	0.1375	0.1154	0.0937	0.0789	0.0682	0.0600	0.0536	0.0484
Transpose	XY	0.1874	0.1785	0.1625	0.1490	0.1328	0.1184	0.1023	0.0900	0.0803	0.0726
	West-first	0.2086	0.2091	0.1897	0.1610	0.1367	0.1184	0.1023	0.0900	0.0803	0.0726
	Negative-first	0.1874	0.1785	0.1625	0.1490	0.1328	0.1184	0.1023	0.0900	0.0803	0.0726
	North-last	0.2086	0.2091	0.1897	0.1610	0.1367	0.1184	0.1023	0.0900	0.0803	0.0726
	Odd-Even	0.1919	0.1903	0.1965	0.1731	0.1406	0.1184	0.1023	0.0900	0.0803	0.0726
Complement	XY	0.2499	0.2499	0.2499	0.2307	0.1875	0.1579	0.1363	0.1200	0.1071	0.0968
	West-first	0.1150	0.1142	0.1052	0.1157	0.1153	0.1150	0.1284	0.1200	0.1071	0.0967
	Negative-first	0.1118	0.1105	0.1170	0.1147	0.1082	0.1215	0.1235	0.1085	0.0970	0.0968
	North-last	0.1459	0.1446	0.1463	0.1460	0.1428	0.1364	0.1278	0.1200	0.1071	0.0968
	Odd-Even	0.1326	0.1330	0.1323	0.1318	0.1325	0.1308	0.1278	0.1200	0.1071	0.0968

**Table 7:** Average latency (in cycles) for a 4 × 4 Torus topology using four arbitration policies under different traffic patterns.

Traffic Pattern	Arbitration Policy	Operating Frequency (MHz)|Offered traffic (normalized)									
		10|1.00	20|0.50	30|0.33	40|0.25	50|0.20	60|0.16	70|0.14	80|0.12	90|0.11	100|0.10
Uniform	Static	15,794.1	6325.1	967.8	76.8	50.3	46.2	43.6	42	41.1	40.5
	Rotative	14,986.1	5513.3	288.3	56.4	48.6	44.7	42.7	41.4	40.5	39.9
	Random	15,063.0	5621.2	172.6	56.5	48.4	44.8	42.7	41.4	40.6	40.0
	Round-Robin	15,193.3	5679.6	621.7	57.5	48.7	44.9	42.7	41.5	40.5	40.0
Bit-reversal	Static	23,346.5	7302.5	8943.7	17.5	17.5	17.5	17.5	17.5	17.5	17.5
	Rotative	30,262.6	12,903.0	7805.4	1503.6	18.0	17.5	17.5	17.5	17.5	17.5
	Random	31,817.6	14,515.5	8398.1	1666.0	18.0	17.5	17.5	17.5	17.5	17.5
	Round-Robin	34,298.3	19,423.6	9121.2	17.5	17.5	17.5	17.5	17.5	17.5	17.5
Perfect Shuffle	Static	10,012.9	13.9	13.9	13.9	13.9	13.9	13.9	13.9	13.9	13.9
	Rotative	10,012.9	13.9	13.9	13.9	13.9	13.9	13.9	13.9	13.9	13.9
	Random	10,012.9	13.9	13.9	13.9	13.9	13.9	13.9	13.9	13.9	13.9
	Round-Robin	10,012.9	13.9	13.9	13.9	13.9	13.9	13.9	13.9	13.9	13.9
Butterfly	Static	35,013.7	12,519.8	14,016.2	17.5	17.5	17.5	17.5	17.5	17.5	17.5
	Rotative	57,414.6	21,719.9	14,017.4	17.5	17.5	17.5	17.5	17.5	17.5	17.5
	Random	57,269.2	21,675.5	14,018.6	17.5	17.5	17.5	17.5	17.5	17.5	17.5
	Round-Robin	70,013.5	43,765.0	17,516.5	17.5	17.5	17.5	17.5	17.5	17.5	17.5
Transpose	Static	17,512.0	3637.3	5483.8	16.0	16.0	16.0	16.0	16.0	16.0	16.0
	Rotative	24,512.9	9147.6	5483.9	16.0	16.0	16.0	16.0	16.0	16.0	16.0
	Random	24,524.8	9121.6	5498.4	16.0	16.0	16.0	16.0	16.0	16.0	16.0
	Round-Robin	26,262.8	12,697.5	6851.0	16.0	16.0	16.0	16.0	16.0	16.0	16.0
Complement	Static	8762.0	13.0	13.0	13.0	13.0	13.0	13.0	13.0	13.0	13.0
	Rotative	8762.0	13.0	13.0	13.0	13.0	13.0	13.0	13.0	13.0	13.0
	Random	8762.0	13.0	13.0	13.0	13.0	13.0	13.0	13.0	13.0	13.0
	Round-Robin	8762.0	13.0	13.0	13.0	13.0	13.0	13.0	13.0	13.0	13.0

**Table 8:** Jitter for a 4 × 4 Torus topology using four arbitration policies under different traffic patterns.

Traffic Pattern	Arbitration Policy	Operating Frequency (MHz)|Offered traffic (normalized)									
		10|1.00	20|0.50	30|0.33	40|0.25	50|0.20	60|0.16	70|0.14	80|0.12	90|0.11	100|0.10
Uniform	Static	9255.3	4627.0	1980.2	91.3	34.2	30.5	27.7	26.0	25.2	24.6
	Rotative	8745.8	3813.9	492.3	36.8	30.2	26.9	25.2	24.3	23.6	23.2
	Random	8790.9	3873.9	264.6	36.9	29.9	27.1	25.4	24.4	23.8	23.2
	Round-Robin	8860.5	4086.2	815.1	38.0	30.1	27.1	25.5	24.6	23.9	23.5
Bit-reversal	Static	5774.1	20,218.7	12,535.4	3.6	3.6	3.6	3.6	3.6	3.6	3.6
	Rotative	12,236.9	15,087.1	12,641.6	6218.7	3.7	3.6	3.6	3.6	3.6	3.6
	Random	13,137.8	16,415.8	13,253.3	4149.8	3.7	3.6	3.6	3.6	3.6	3.6
	Round-Robin	12,594.4	12,655.8	5119.3	3.6	3.6	3.6	3.6	3.6	3.6	3.6
Perfect Shuffle	Static	2474.7	3.1	3.1	3.1	3.1	3.1	3.1	3.1	3.1	3.1
	Rotative	2474.7	3.1	3.1	3.1	3.1	3.1	3.1	3.1	3.1	3.1
	Random	2474.7	3.1	3.1	3.1	3.1	3.1	3.1	3.1	3.1	3.1
	Round-Robin	2474.7	3.1	3.1	3.1	3.1	3.1	3.1	3.1	3.1	3.1
Butterfly	Static	8664.7	31,682.6	17,993.6	1.5	1.5	1.5	1.5	1.5	1.5	1.5
	Rotative	22,388.5	30,757.4	17,993.2	1.5	1.5	1.5	1.5	1.5	1.5	1.5
	Random	22,605.4	30,743.9	17,991.9	1.5	1.5	1.5	1.5	1.5	1.5	1.5
	Round-Robin	17,320.6	10,825.4	4330.1	1.5	1.5	1.5	1.5	1.5	1.5	1.5
Transpose	Static	4330.1	12,960.9	9851.8	4.2	4.2	4.2	4.2	4.2	4.2	4.2
	Rotative	10,711.1	13,430.6	9849.1	4.2	4.2	4.2	4.2	4.2	4.2	4.2
	Random	10,667.0	13,392.2	9770.8	4.2	4.2	4.2	4.2	4.2	4.2	4.2
	Round-Robin	11,110.9	12,500.1	5712.6	4.2	4.2	4.2	4.2	4.2	4.2	4.2
Complement	Static	2165.1	0.0	0.0	0.0	0.0	0.0	0.0	0.0	0.0	0.0
	Rotative	2165.1	0.0	0.0	0.0	0.0	0.0	0.0	0.0	0.0	0.0
	Random	2165.1	0.0	0.0	0.0	0.0	0.0	0.0	0.0	0.0	0.0
	Round-Robin	2165.1	0.0	0.0	0.0	0.0	0.0	0.0	0.0	0.0	0.0

**Table 9:** Throughput (normalized) for a 4 × 4 Torus topology using four arbitration policies under different traffic patterns.

Traffic Pattern	Arbitration Policy	Operating Frequency (MHz)|Offered traffic (normalized)									
		10|1.00	20|0.50	30|0.33	40|0.25	50|0.20	60|0.16	70|0.14	80|0.12	90|0.11	100|0.10
Uniform	Static	0.3295	0.3295	0.2910	0.2306	0.1874	0.1578	0.1363	0.1200	0.1071	0.0967
	Rotative	0.3398	0.3406	0.2977	0.2306	0.1874	0.1578	0.1363	0.1200	0.1071	0.0967
	Random	0.3390	0.3389	0.2986	0.2306	0.1874	0.1578	0.1363	0.1200	0.1071	0.0967
	Round-Robin	0.3370	0.3377	0.2948	0.2306	0.1874	0.1578	0.1363	0.1200	0.1071	0.0967
Bit-reversal	Static	0.1875	0.1098	0.1050	0.1730	0.1406	0.1184	0.1023	0.0900	0.0803	0.0726
	Rotative	0.2249	0.2159	0.1892	0.1682	0.1406	0.1184	0.1023	0.0900	0.0803	0.0726
	Random	0.2141	0.2058	0.1845	0.1684	0.1406	0.1184	0.1023	0.0900	0.0803	0.0726
	Round-Robin	0.2187	0.2098	0.1937	0.1730	0.1406	0.1184	0.1023	0.0900	0.0803	0.0726
Perfect Shuffle	Static	0.4374	0.3749	0.2625	0.2019	0.1640	0.1381	0.1193	0.1050	0.0937	0.0847
	Rotative	0.4374	0.3749	0.2625	0.2019	0.1640	0.1381	0.1193	0.1050	0.0937	0.0847
	Random	0.4374	0.3749	0.2625	0.2019	0.1640	0.1381	0.1193	0.1050	0.0937	0.0847
	Round-Robin	0.4374	0.3749	0.2625	0.2019	0.1640	0.1381	0.1193	0.1050	0.0937	0.0847
Butterfly	Static	0.0750	0.1249	0.1250	0.1154	0.0937	0.0789	0.0682	0.0600	0.0536	0.0484
	Rotative	0.1249	0.1249	0.1250	0.1154	0.0937	0.0789	0.0682	0.0600	0.0536	0.0484
	Random	0.1243	0.1244	0.1250	0.1154	0.0937	0.0789	0.0682	0.0600	0.0536	0.0484
	Round-Robin	0.1250	0.1250	0.1250	0.1154	0.0937	0.0789	0.0682	0.0600	0.0536	0.0484
Transpose	Static	0.2500	0.2320	0.2000	0.1731	0.1406	0.1184	0.1023	0.0900	0.0804	0.0726
	Rotative	0.2498	0.2320	0.1999	0.1731	0.1406	0.1184	0.1023	0.0900	0.0804	0.0726
	Random	0.2477	0.2265	0.1974	0.1731	0.1406	0.1184	0.1023	0.0900	0.0804	0.0726
	Round-Robin	0.2499	0.2321	0.2000	0.1731	0.1406	0.1184	0.1023	0.0900	0.0804	0.0726
Complement	Static	0.5000	0.4286	0.3000	0.2308	0.1875	0.1579	0.1364	0.1200	0.1071	0.0968
	Rotative	0.5000	0.4286	0.3000	0.2308	0.1875	0.1579	0.1364	0.1200	0.1071	0.0968
	Random	0.5000	0.4286	0.3000	0.2308	0.1875	0.1579	0.1364	0.1200	0.1071	0.0968
	Round-Robin	0.5000	0.4286	0.3000	0.2308	0.1875	0.1579	0.1364	0.1200	0.1071	0.0968

**Table 10:** Average latency (in cycles) for a 4 × 2 × 2 Mesh topology using seven memorization schemes under Uniform traffic.

Memorization scheme	Operating Frequency (MHz)|Offered traffic (normalized)									
	10|1.00	20|0.50	30|0.33	40|0.25	50|0.20	60|0.16	70|0.14	80|0.12	90|0.11	100|0.10
4-flit input buffer	23,312.0	10,125.0	371.5	58.7	50.0	46.0	43.6	42.2	41.1	40.5
8-flit input buffer	18,133.1	4969.9	63.7	49.1	44.7	42.6	41.4	40.6	40.1	29.7
16-flit input buffer	16,310.1	3160.0	50.9	44.8	42.5	41.3	40.5	40.0	39.6	39.3
32-flit input buffer	16,182.4	2932.3	47.9	43.9	42.0	41.0	40.2	39.8	39.4	39.1
4-flit input and 4-flit output buffers	18,868.7	5655.5	70.1	52.8	47.5	45.1	43.7	42.8	42.2	41.7
8-flit input and 8-flit output buffers	16,511.7	3245.7	54.4	47.0	44.5	43.3	42.5	41.9	41.5	41.2
16-flit input and 16-flit output buffers	16,277.8	3006.7	49.8	45.9	44.0	42.9	42.2	41.8	41.4	41.1

**Table 11:** Jitter for a 4 × 2 × 2 Mesh topology using seven memorization schemes under Uniform traffic.

Memorization scheme	Operating Frequency (MHz)|Offered traffic (normalized)									
	10|1.00	20|0.50	30|0.33	40|0.25	50|0.20	60|0.16	70|0.14	80|0.12	90|0.11	100|0.10
4-flit input buffer	5811.1	3017.8	485.4	34.4	27.9	25.6	24.4	23.7	23.2	22.9
8-flit input buffer	4629.2	2615.6	38.3	28.1	25.6	24.4	23.7	23.2	23.0	22.7
16-flit input buffer	4306.3	2699.4	29.4	26.0	24.7	23.9	23.5	23.1	22.9	22.6
32-flit input buffer	4269.1	2766.6	27.8	25.6	24.5	23.8	23.4	23.0	22.8	22.6
4-flit input and 4-flit output buffers	4809.4	2531.1	40.3	29.2	26.6	24.7	23.8	23.2	22.9	22.7
8-flit input and 8-flit output buffers	4342.8	2717.2	30.3	26.2	24.7	23.9	23.4	23.0	22.8	22.5
16-flit input and 16-flit output buffers	4262.0	2737.3	27.9	25.7	24.5	23.8	23.4	23.0	22.8	22.6

**Table 12:** Throughput for a 4 × 2 × 2 Mesh topology using seven memorization schemes under Uniform traffic.

Memorization scheme	Operating Frequency (MHz)|Offered traffic (normalized)									
	10|1.00	20|0.50	30|0.33	40|0.25	50|0.20	60|0.16	70|0.14	80|0.12	90|0.11	100|0.10
4-flit input buffer	0.3216	0.3203	0.2971	0.2303	0.1871	0.1575	0.1361	0.1197	0.1069	0.0966
8-flit input buffer	0.3690	0.3681	0.2994	0.2303	0.1871	0.1576	0.1361	0.1197	0.1069	0.0966
16-flit input buffer	0.3865	0.3869	0.2994	0.2303	0.1871	0.1576	0.1361	0.1197	0.1069	0.0966
32-flit input buffer	0.3882	0.3888	0.2994	0.2303	0.1871	0.1576	0.1361	0.1197	0.1069	0.0966
4-flit input and 4-flit output buffers	0.3607	0.3608	0.2995	0.2303	0.1872	0.1576	0.1361	0.1198	0.1069	0.0966
8-flit input and 8-flit output buffers	0.3852	0.3852	0.2994	0.2303	0.1872	0.1576	0.1361	0.1198	0.1069	0.0966
16-flit input and 16-flit output buffers	0.3883	03875	0.2994	0.2303	0.1871	0.1576	0.1361	0.1197	0.1069	0.0966

## 2 Experimental design, materials, and methods

The experiments used synthetic traffic generators to inject packets into the network. Traffic generation and analysis employed the model proposed in Ref. [4] with the discard of the first packages delivered to avoid the systematic bias of the simulation, according to the method presented by Ref. [5]. The simulations applied six spatial traffic distributions, including Uniform, Bit-reversal, Perfect Shuffle, Butterfly, Transpose, and Complement [6]. In spatial distributions in which a source node can generate multiple communication flows to different destinations (e.g., Uniform traffic), their packets were generated and injected in random order with the use of the random number generator of the C++ Standard Template Library (STL), which relies on a uniform discrete distribution. This generator utilizes a seed computed in the front-end, and this seed is derived from the current simulation start time. In permutation-based distributions, when the destination node of a communication flow was the source node itself, the traffic generator did not generate the corresponding packets. Regarding the temporal distribution, each communication flow consisted of injecting 128-bit packets into 32-bit data links at a constant injection rate of 320 Mbps.

As shown in the tables presented above, the experiments evaluated the performance of six on-chip communication architectures, including Bus, Crossbar, and four NoC topologies (Chordal Ring, 2D Mesh, 3D Mesh, and 2D Torus). The experiments did not consider all the architectures for each spatial distribution. We did not evaluate the permutation-based distributions on the Bus as traffic does not depend on the destination addresses. We did not also consider NoC topologies in which a deadlock condition was reached because the routing algorithm was not able to avoid it. In addition to the topologies, the experiments also evaluated five routing alternatives in a 2D Mesh, four arbitration policies in a 2D Torus, and seven memorization schemes in a 3D Mesh.

We have configured the simulations to run until the delivery of 100,000 packets. The first 40,000 packets (40%) were discarded from the analysis to reduce sampling bias relative to the network warm-up period. It was not necessary to discard packets at the end of the simulation (drain period) as the simulator did not stop generating packages until the stop condition was reached.

The experiments were performed under different operating frequencies. As the data link width equals 32 bits and the injection rated is constant (320 Mbps), each operating frequency (e.g., 50 MHz) corresponds to a specific offered traffic (e.g., 0.20), as it is shown in the header of each table above.

The metrics used in the experiments were the average packet latency, jitter (defined by the standard deviation of packet latencies), and the throughput (which expresses the accepted traffic). These data are presented in the tables above and can be used as a reference for researches on on-chip communication architectures.
